# Comparative metabolite profiling of drought stress in roots and leaves of seven Triticeae species

**DOI:** 10.1186/s12864-017-4321-2

**Published:** 2017-12-15

**Authors:** Naimat Ullah, Meral Yüce, Z. Neslihan Öztürk Gökçe, Hikmet Budak

**Affiliations:** 1 Faculty of Engineering and Natural Sciences, Sabanci University, 34956 Istanbul, Turkey; 2 Nanotechnology Research and Application Centre, Sabanci University, 34956 Istanbul, Turkey; 3 Ayhan Sahenk Faculty of Agricultural Sciences and Technologies, Nigde Omer Halisdemir University, 51240 Nigde, Turkey; 4 Department of Plant Science and Plant Pathology, Montana State University, Bozeman, MT USA

**Keywords:** Triticeae, Drought stress, Metabolome, Systems biology, Organic acids, Biochemical pathway, Plant genomics

## Abstract

**Background:**

Drought is a lifestyle disease. Plant metabolomics has been exercised for understanding the fine-tuning of the potential pathways to surmount the adverse effects of drought stress. A broad spectrum of morphological and metabolic responses from seven Triticeae species including wild types with different drought tolerance/susceptibility level was investigated under control and water scarcity conditions.

**Results:**

Significant morphological parameters measured were root length, surface area, average root diameter and overall root development. Principal Component Analysis, Partial Least-Squares-Discriminant Analysis and Hierarchical Cluster Analysis were applied to the metabolomic data obtained by Gas Chromatography-Mass Spectrometry technique in order to determine the important metabolites of the drought tolerance across seven different Triticeae species. The metabolites showing significant accumulation under the drought stress were considered as the key metabolites and correlated with potential biochemical pathways, enzymes or gene locations for a better understanding of the tolerance mechanisms. In all tested species, 45 significantly active metabolites with possible roles in drought stress were identified. Twenty-one metabolites out of forty-five including sugars, amino acids, organic acids and low molecular weight compounds increased in both leaf and root samples of TR39477, IG132864 and Bolal under the drought stress, contrasting to TTD-22, Tosunbey, Ligustica and Meyeri samples. Three metabolites including succinate, aspartate and trehalose were selected for further genome analysis due to their increased levels in TR39477, IG132864, and Bolal upon drought stress treatment as well as their significant role in energy producing biochemical pathways.

**Conclusion:**

These results demonstrated that the genotypes with high drought tolerance skills, especially wild emmer wheat, have a great potential to be a genetic model system for experiments aiming to validate metabolomics–genomics networks.

**Electronic supplementary material:**

The online version of this article (10.1186/s12864-017-4321-2) contains supplementary material, which is available to authorized users.

## Background

Wheat, an outstanding member of Triticeae, attracts worldwide attention among many other crops, particularly in the face of increasing world population and the global climate change [1, 2]. Drought affects more than 70% of arable lands around the world, and the drought stress-related yield loss has gained considerable attention in recent years as agricultural activities have been extended to less fertile or infertile fields to meet the growing food demand. As a result, the enhancement of the drought tolerance in crops, i.e. the survival of the plant under limited water conditions with the maintenance of high productivity, has become a key challenge for today’s wheat agronomists and plant geneticists [2, 3]. Although several genes involved in the mechanism have been identified over the past few decades [3, 4], the drought stress response is still a complex phenomenon with several key factors that have yet to be investigated. Various omics fields including physiology, molecular biology, transcriptomics and metabolomics have been used [5] in order to clarify the abiotic stress mechanisms and reveal the potential metabolic pathways that can be manipulated to surmount the adverse effects of the limited water conditions. Plant metabolomics has been extensively exercised for understanding the fine-tuning of the metabolism in response to several abiotic stresses [6–8], especially to understand the production of compatible solutes including proline, asparagine, polyamines, glycine betaine, γ-amino-N-butyric acid (GABA), raffinose, trehalose, sucrose, and polyols [9]. Drought-inducible nature of some of these compatible solutes was proven in an integrated metabolomic and transcriptomic studies with Arabidopsis and grapevine [10, 11]. An untargeted metabolomic analysis of wild and transgenic *Nicotiana langsdorffii* plants with high-performance liquid chromatography separation (HPLC) coupled to high-resolution mass spectrometry (HRMS) enabled the identification of more than 200 metabolites upon exposure to different abiotic stresses (high temperature, water deficit, and high chromium concentrations) [12]. The same study revealed the drought-specific accumulation in several antioxidants including polyamines [12]. A targeted metabolite profiling using liquid chromatography-tandem mass spectrometry (LC-MS) for comparison of onion doubled haploid (*Allium cepa* L., DHC), shallot doubled haploid (*A. cepa* L. Aggregatum group, DHA), and F_1_ hybrid identified 113 targeted metabolites linked to abiotic stress response indicating different levels of stress tolerance in these genotypes [13]. A very recent study with time-of-flight mass spectrometry in the highly drought tolerant plant *Caragana korshinskii* leaf, stem, root collar, and root upon drought stress identified several hundred metabolites with differing levels depending on the organ, with a well-conserved abundance of various small carbohydrates having a potential to act as compatible solutes or antioxidants [14]. The leaves and roots of two barley (*Hordeum vulgare* L.) with contrasting drought tolerance were identified 121 drought-responsive proteins in leaves and 182 in roots of both genotypes with two-dimensional electrophoresis (2D–PAGE) combined with matrix-assisted laser desorption time of flight mass spectrometry (MALDI-TOF) [15]. Most of the proteins identified were of stress-responsive in nature with photosynthesis and carbon metabolism [15].

The summarized studies indicated plant, genotype, organ, developmental stage and desiccation level specific strategy to cope with drought stress. To go further in these observations, in this study, low-molecular-weight drought stress-responsive metabolites in the root and leave samples of seven wild and domesticated wheat relatives were revealed by a gas chromatography-mass spectrometry (GC-MS) based comparative metabolomics approach. The Triticeae species, widely used and well characterized for their drought tolerance index in our previous reports, were *Triticum turgidum ssp. dicoccoides* genotype TR39477 (TR), *Triticum monococcum ssp. monococcum* genotype IG132864 (TM), *Triticum aestivum ssp. aestivum* genotype Bolal (TA), *Aegilops tauschii var. Meyeri* (A), *Aegilops speltoids var. Ligustica* (AS), *Triticum turgidum ssp. dicoccoids* genotype TTD-22 (TD) and *Triticum aestivum ssp. aestivum* genotype Tosunbey (Tosun) [1, 4, 16, 17]. The metabolic content of these Triticeae species was compared to reveal the effects of drought stress on the metabolomic level. The mechanisms of plant adaptation to drought stress were also observed through morphological examination of the sample roots. The outcomes of this study provide a valuable source for metabolome of modern and wild wheat species, which can eventually contribute to the future genetic and metabolomic studies of the domesticated crops.

## Methods

### Plant growth conditions and drought stress treatment

Wild and domesticated wheat genotypes from different ploidy levels that our group has used in several previous research focusing on their drought stress response in transcriptome and/or miRNA expression levels were combined for comparison [1, 4, 16, 17]. A list of the species used in this study was presented in Table 1.

**Table 1:** List of Triticeae species used in the study

Species	Common Name	Genome	Genotype	Abbreviations	
				Drought	Control
*Aegilops speltoides ssp. speltoids*	Goatgrass	BB	Ligustica	AS	ASC
*Aegilops tauschii ssp. tauschii*	Goatgrass	DD	Meyeri	A	AC
*Triticum turgidum ssp. dicoccoides*	Wild emmer	AABB	TR39477	TR	TRC
*Triticum turgidum* ssp. *dicoccoides*	Wild emmer	AABB	TTD-22	TD	TDC
*Triticum monococcum ssp. monococcum*	Einkorn domesticated	AA	IG132864	TM	TMC
*Triticum aestivum ssp. aestivum*	Bread wheat	AABBDD	Bolal	TA	TAC
*Triticum aestivum* ssp. *aestivum*	Bread wheat	AABBDD	Tosunbey	Tosun	TosunC

“Drought” refers to the samples that are exposed to drought stress. “Contol” refers to the samples that are not exposed to drough stress

The seeds used in this study were obtained from Ministry of Food, Agriculture and Livestock (Turkey) GenBanks. The seeds were surface sterilised and pre-germinated in Petri dishes (20 plants from each genotype) for 21 days at 4 °C in the dark. Three plastic pots (2 kg) were grown under controlled conditions (16 h photoperiod, temperature 24/22 °C, relative humidity 60%, and photon flux density of 600–700 μmol m^−2^ s^−1^) for each genotype for each treatment, and each pot contained ten plants un. Plants at the stage of three leaves were watered to 80%, 50% and 30% field capacity (FC) that were served as control, mild-stressed and the drought-treated samples, respectively [6]. Maintenance of the FC was assured by daily weighing of the pots, replacing the water lost by transpiration and evaporation from the pot and the plant surface. After 16 days of further growth, three biological replicates from each genotype under control and drought stress conditions were sampled (for each replicate with six seedlings, an equal amount of sample from randomly selected five individual plants were pooled). All leaf and root samples were immediately frozen in liquid nitrogen and stored at −80 °C until metabolite extraction process.

### Measurement of root morphological parameters

Three plants with full roots from each genotype under control and drought stress conditions were collected after 16 days of treatment, washed and dried to analyse root morphological parameters. The root length, average root diameter, surface area, number and length of lateral roots, number of tips, number of forks and crossings (overlapping parts) were measured with WinRHIZO 4.1 system (Regent Instruments Inc.; Quebec, Canada) [7–9]. Lateral root initiations and the diameter of primary roots were measured under optical light microscope illumination (10X–lense) [10, 11].

### Extraction of metabolites and GC-MS measurements

Standard mixtures used for the optimisation of GC-MS studies were prepared in 1000 μg/ml methanol and stored at −20 °C. Working standard solution was diluted up to 50 μg/ml from the main stock solution. Polar metabolites were extracted with 350 μl of 100% methanol and suspended in 20 μl of internal polar standard (Ribitol; 0.2 mg/ml in water) [12]. The mixture was incubated at 70 °C for 15 min and mixed with 1 volume of distilled water. Chloroform (300 μl) was added to the mixture to separate polar and non-polar metabolites, followed by centrifugation at 14000 rpm for 10 min. The supernatant was taken and washed again with chloroform. Aliquots of the leaf and root polar phases (100 μl and 5 μl) were used for the analysis of high and low abundance metabolites while the non-polar phase was discarded. All aliquots were dried under vacuum, re-dissolved and derivatized at 37 °C for 2 h in methoxy-amine-hydrochloride (40 μl of 30 mg/ml in pyridine). Trimethylsilylation was performed at 37 °C for 30 min with N-methyl-N-[trimethylsilyl] trifluoroacetamide (70 μl; MSTFA) [13].

GC-MS-QP2010 Ultra Gas Chromatograph Mass Spectrometer with an AOC-20i auto-injector GC Ultra and a DSQ quadrupole MS (SHIMADZU Corporation, Tokyo, 101–8448, Japan) was used for metabolite profiling. The MS was tuned according to the manufacturer’s recommendations using tris-(perfluorobutyl)-amine (CF43). GC was performed on a 30-m MDN-35 capillary column with 0.25 mm inner diameter and 0.25 μm film thickness (Varian Inc., Victoria, Australia). The injection temperature was set at 230 °C, the MS transfer line at 280 °C, and the ion source at 250 °C. Helium gas at 99.99% purity was used as the carrier gas with 1 ml/min flow rate. The analysis was performed under the following oven temperature program: injection at 70 °C followed by 1 °C/min ramp to 76 °C, and then by 6 °C/min to 330 °C, finishing with 10 min isothermal at 330 °C. The samples obtained were injected into the GC-MS column in the splitless mode, using the hot needle technique. The GC-MS system was then temperature-equilibrated for 1 min at 70 °C before injection of the next sample [14].

### GC-MS data analysis

GC-MS data was acquired through Advanced Scanning Speed Protocol (ASSPTM) integrated into the GC-MS-QP2010 Ultra at a speed of 20,000 μ/s and 100 Hz. Both chromatograms and mass spectra of the eluted compounds were identified using the AMDIS program (version 2.72) with the mass spectral reference NIST library comprised of the spectra of 191,436 general compounds, and Wiley Registry of Mass Spectral Library accompanied by the corresponding structural information, enabling the discovery of new components as well as the targeted ones. Authentic standards were used to analyse and verify all matching spectra [15]. The pseudo peaks originating from the internal standards or caused by noise, column and derivatization procedure, were removed from the dataset. The peaks with similarity index higher than 70% were considered effective metabolites in the experiments, while those with a similarity index less than 70% similarity index were removed from the data. Following the deconvolution of the resulting chromatograms, metabolic compounds including amino acids, organic acids, and sugars were identified. Each metabolic compound was given a specific trace to be used in the quantification [16]. The resulting peak areas were normalised to the area of a specific trace of the internal standard resulting in relative response ratios, which were further normalised by the fresh weight of each sample.

The complete metabolomics data were mean-centred for Principal Component Analysis (PCA) and Partial Least-Squares-Discriminant Analysis (PLS-DA). Hierarchical Cluster Analysis (HCA) was performed using Cluster (version 3.0). Total explained variance (R^2^) and predictability (Q^2^) values were extracted from the metabolomic data by using unit variance scaling method. A two-sample t-test was applied to find the level of significance between the metabolites, and the inter-connection between significantly altered metabolites was analysed by using R software. The Cytoscape software was used to reveal metabolite-metabolite interaction and gene-metabolite networks by integrating the data [17–19]. *P* values less than 0.05 were considered statistically significant and less than 0.01 as highly significant.

### Identification and location of genes in wheat genome

For the validation of data, the full-length complementary DNA (FL-cDNA) and potential wheat genes encoding the two enzymes in WGSS and root transcriptome data were used to extract orthologues of the genes encoding enzymes of biochemical pathways responsible for the biosynthesis of drought-specific metabolites. The sequencing data analysed during the current study is publicly available in the recent report by Cagirici et al. [20]. Later, TBLASTX search (e-value < 3e^−106^) was adopted by using annotated rice orthologous cDNA sequences to identify corresponding wheat FL-cDNAs from the Chinese spring collection [21] and the transcriptome data [22]. Finally, BLASTN search was performed against the Wheat Genome Survey Sequences (WGSS). The wheat FL-cDNA sequences were used as a query in BLASTN search against the wheat genome survey sequence (WGSS), and the chromosomal locations were identified based on a threshold value of 85% sequence identity.

## Results

### Morphological responses of roots to the drought stress

Different levels of drought stress including control (80% FC), mild (50% FC) and severe drought stress (30% FC) were applied to the samples to investigate the changes in the morphology of the roots and metabolic content of seven different genotypes. Interestingly, no noticeable morphological differences were observed between the control samples and individuals exposed to the 50% FC for 16 days, whereas 30% FC caused severe effects on the morphology and physiology of the drought-sensitive plants. Therefore, 30% FC was chosen to compare the morphological and metabolic responses of the samples from different ploidy levels.

Morphological responses of the roots to the drought stress from all seven genotypes (Ligustica, Meyeri, TR39477, TTD-22, IG132864, Bolal and Tosunbey) were found statistically significant with a *p*-value less than 0.05 as presented in Table 2. The average root length and surface area were increased in TR39477, IG132864 and Bolal while few to no lateral root formation and reduction in the diameters of the primary and secondary roots were observed. Morphological changes were practically reverse in the sensitive genotypes such as TTD-22, Tosunbey, Ligustica, and Meyeri. For example, the mean values of root length in TR39477, IG132864 and Bolal increased from 32.4 cm, 33.6 cm and 37.9 cm to 51.5 cm, 54.1 cm and 61.6 cm, respectively after the drought stress induction, whereas the mean values of root length in TTD-22, Meyeri, Ligustica and Tosunbey decreased from 32.9 cm, 32.1 cm, 31.7 cm and 32.0 cm to 11.7 cm, 18.9 cm, 16.7 cm and 14.7 cm, respectively (Additional file 1: Figure S1a-1b). Similar results were obtained related to surface area parameter, for example, the mean value of the surface area in TR39477 increased from 81.2 cm^2^ to 119.2 cm^2^, whereas it decreased in the drought-sensitive TTD-22 from 61.6 cm^2^ to 36.3 cm^2^. The diameters of the primary and secondary roots were found to be smaller in the drought-resistant genotypes (mean value, 13.8 μm) than the same genotypes under standard water conditions (average value, 19.17 μm) (Additional file 1: Figure S1 c-f). Other morphological parameters including the number of tips and forks were less common in the drought stress tolerant wheat genotypes as compared to the well-watered plants of the same cultivars.

**Table 2:** Morphological parameters of the roots measured for all genotypes by using WinRHIZO system

Species	Treatment	ARA	ARW	ARH	RL	PA	SA	AvD	LPV	RV	Tips	t-test
*Aegilops speltoides* ssp. *speltoids*	Control	54.80	36.36	94.54	32.47	50.24	84.44	10.15	24.55	21.70	32	0.016
	Drought	49.66	77.84	72.18	51.52	54.39	109.26	23.82	83.17	40.05	18	0.001
*Aegilops tauschii* ssp. *tauschii*	Control	60.20	22.76	11.67	32.17	45.41	71.55	39.46	39.99	10.62	31	0.014
	Drought	10.72	27.15	21.17	18.97	42.71	44.33	60.14	14.10	34.59	20	0.010
*Triticum turgidum* ssp. *dicoccoides*	Control	77.34	10.51	12.39	33.64	60.05	81.27	17.74	42.18	14.58	11	0.012
	Drought	10.03	45.46	93.87	54.12	80.97	119.20	13.93	60.36	17.13	10	0.037
*Triticum turgidum* ssp. *dicoccoides*	Control	15.22	43.81	14.44	32.99	43.78	61.64	98.22	29.64	13.84	62	0.017
	Drought	41.79	82.78	78.48	11.70	23.05	36.36	68.95	19.10	33.78	25	0.010
*Triticum monococcum* ssp. *monococcum*	Control	82.80	97.62	13.70	31.76	47.23	81.53	19.97	98.72	88.59	25	0.022
	Drought	88.67	38.59	14.97	16.78	62.25	28.37	10.62	28.84	25.16	30	0.012
*Triticum aestivum* ssp. *aestivum*	Control	19.71	21.78	36.32	37.97	94.23	76.41	23.60	46.97	34.52	13	0.025
	Drought	70.98	10.32	12.28	61.63	38.02	120.37	48.78	18.04	25.51	27	0.001
*Triticum aestivum* ssp. *aestivum*	Control	40.85	40.94	61.47	32.21	51.92	71.46	12.12	31.44	88.59	18	0.011
	Drought	52.96	18.52	10.70	14.75	74.33	53.38	19.97	11.04	25.16	22	0.006

ARA (cm^2^) Analysed Region Area (cm^2^); ARW (cm) Analysed Region Width (cm); ARH (cm) Analysed Region Height (cm); RL (cm) Root Length (cm); PA (cm^2^) Projected Area (cm^2^); SA (cm^2^); Surface Area (cm^2^); AvD (mm)

### Metabolic profile analysis upon control and drought stress treatments

**Fig. 1:**
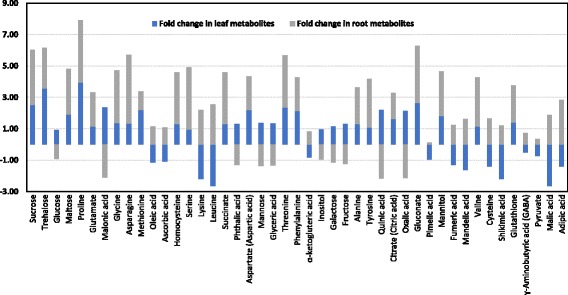
The fold changes in the concentrations of each metabolite upon drought stress were calculated for leaf and root samples using GC-MS data (GC-MS spectra for each genotype and their corresponding values were presented in Additional file 1: Figures S2–5 and Tables S1–2)

Metabolites were extracted from the leaf and root tissue samples in triplicates from all seven Triticeae species for each of the four experimental groups, including drought stress treated leaves (DSL), drought stress treated roots (DSR), control leaves (CL) and control roots (CR). All four groups presented distinct chromatographic patterns, and 45 metabolic compounds were differentially accumulated, embracing amino acids, organic acids, sugars, organic compounds and organic antioxidants and compatible solutes as presented in Fig. 1. Corresponding GC-MS spectra for each genotype and the calculations of fold changes were presented in the Additional file 1: Figures S2–5 and Tables S1–2.

The amount of 21 metabolites out of 45 including sugars or its derivatives (sucrose, trehalose, mannitol and maltose), amino acids (proline, glutamate, alanine, glycine, asparagines, methionine, threonine, phenylalanine, homocysteine, serine, valine and tyrosine), organic acids (succinate, citrate, aspartate and gluconate) and low molecular weight compounds (glutathione) increased in both leaf and root samples of TR39477, IG132864 and Bolal under drought stress, contrasting to TTD-22, Tosunbey, Ligustica and Meyeri samples. The coordinated decrease in the accumulation levels of ɣ-aminobutyric acid (GABA), pyruvate, α-ketoglutarate, was found both in the leaf and root tissue samples of all seven genotypes. The accumulation levels of 10 metabolic compounds including glucose, inositol, galactose, fructose, mannose, glyceric acid, quinic acid, malonic acid, oxalic acid, phthalic acid presented a decrease in the roots of TR39477, IG132864 and Bolal whereas these metabolites (mainly sugars) were present in normal levels in the leaf samples. The remaining four genotypes (TTD-22, Tosunbey, Ligustica, and Meyeri) presented a lower standard of accumulation for glucose, inositol, galactose, fructose, mannose, glyceric acid, quinic acid, malonic acid, oxalic acid, the phthalic acid in the leaf and root samples. On the other hand, accumulation level of the remaining 11 metabolic compounds (pimelic acid, shikimic acid, malic acid, adipic acid, oleic acid, ascorbic acid, fumaric acid, mandelic acid, lysine, leucine, and cysteine) decreased in the leaf samples of TR39477, IG132864 and Bolal compared to the root and control samples whereas they were accumulated in moderate to high levels in the leaf and root tissue samples of TTD-22, Tosunbey, Ligustica and Meyeri genotypes, respectively.

### PCA, PLS-DA and HCA results

**Fig. 2:**
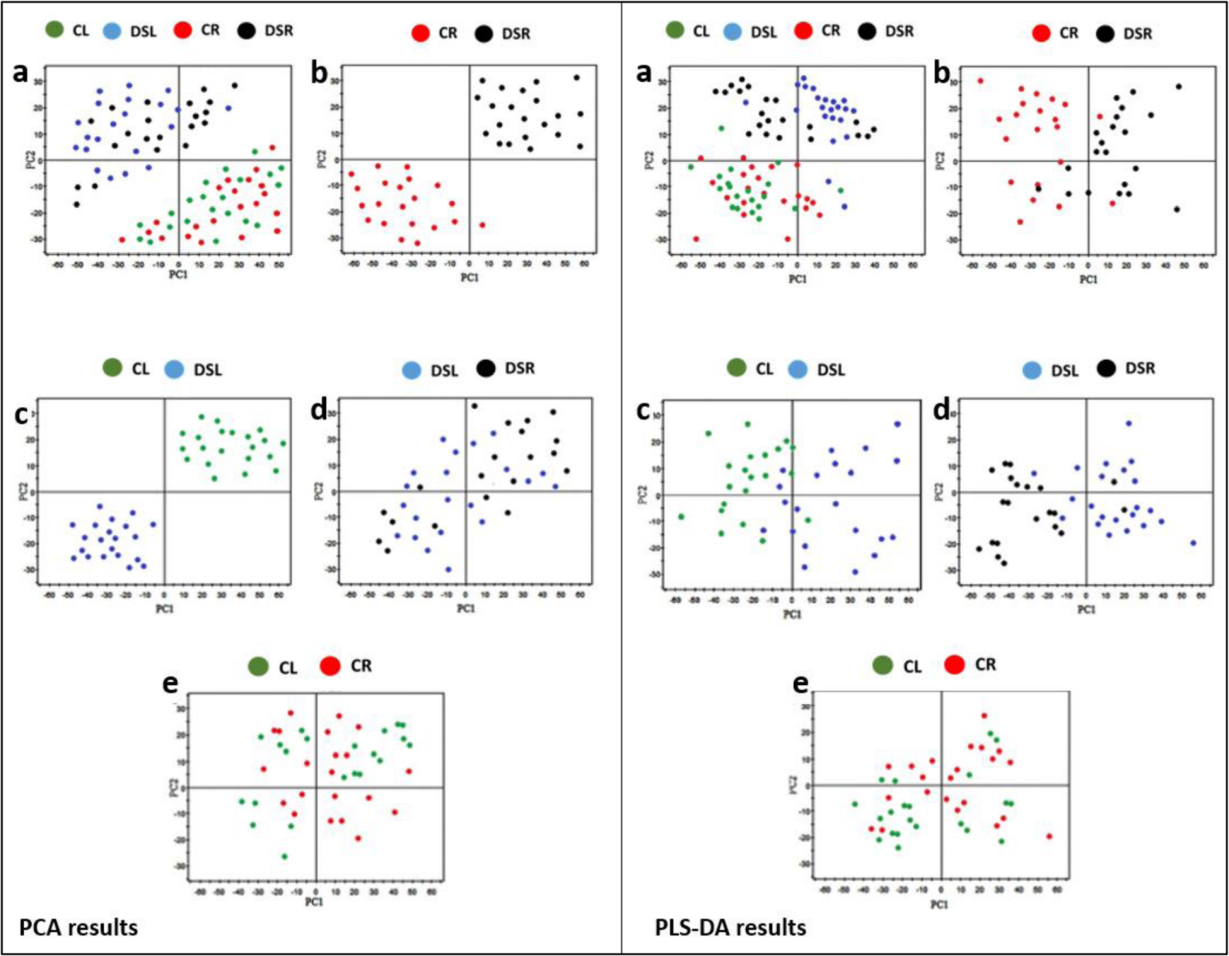
Principal component analysis (PCA) and Partial least squares-discriminate analysis (PLS-DA) score plots for metabolites in wheat leaves and roots under control and drought stress conditions. **a**) score plot for control leaves (CL; green), drought stress leaves (DSL; blue), control roots (CR; red) and drought stress roots (DSR; black) samples, **b**) score plot for CR and DSR samples, **c**) score plot for CL and DSL samples, **d**) PCA score plot for DSL and DSR samples and **e**) PCA score plot for CL and CR samples

PCA was performed to reduce the dimensionality of the metabolomics data generated by GC-MS. The explanation and predictability values measured for the first two Principal Components (PCs) were found 71.2% and 42.6%, respectively (Additional file 1: Table S3). PCA analysis revealed the discriminations between 80% FC and 30% FC samples, but, an overlap was observed between the leaf and root samples (Fig. 2a, left panel). PCA analysis was also applied for each of the remaining three groups including CL vs DSL, CR vs DSR and DSL vs DSR in order to contrast the datasets for better understanding. As given in Fig. 2b-e left panel, a discriminative boundary between every two groups aforementioned was not achievable. Therefore, PLS-DA was applied to classify the observations in the groups by giving the largest predicted indicator variable (Fig. 2a, right panel).

The altered metabolites with significant (*P* < 0.05) and highly significant (*P* < 0.01) fold changes were obtained from the X-loading plots of the PC1 in PLS-DA. Variable importance in the projection (VIP) values was calculated for each altered metabolite and made a cut-off point for all metabolites obtained from the GC-MS analysis. The metabolites that were having VIP values greater than one were considered as the most relevant ones for the drought stress responses. The prediction results were satisfactory when only two principal components were obtained using the data from the control and drought stress-treated samples, whereas both drought stress-treated groups were separated from the control groups along the first principal component, PC1 (Fig. 2b and c, right panel). In addition to the overlapping, DSL, and DSR samples were separated in the PLS-DA score plot with two PCs (Fig. 2d and e, right panel). The comparison among similar treatments such as drought stress treated groups (DSL-DSR) and control groups (CL-CR) presented values 0.482 and 0.461 for R^2^Y whereas 0.375 and 0.058 for Q^2^, respectively (Additional file 1: Table S3), indicating a minor metabolic change between the same treatments as compared to the respective controls.

**Fig. 3:**
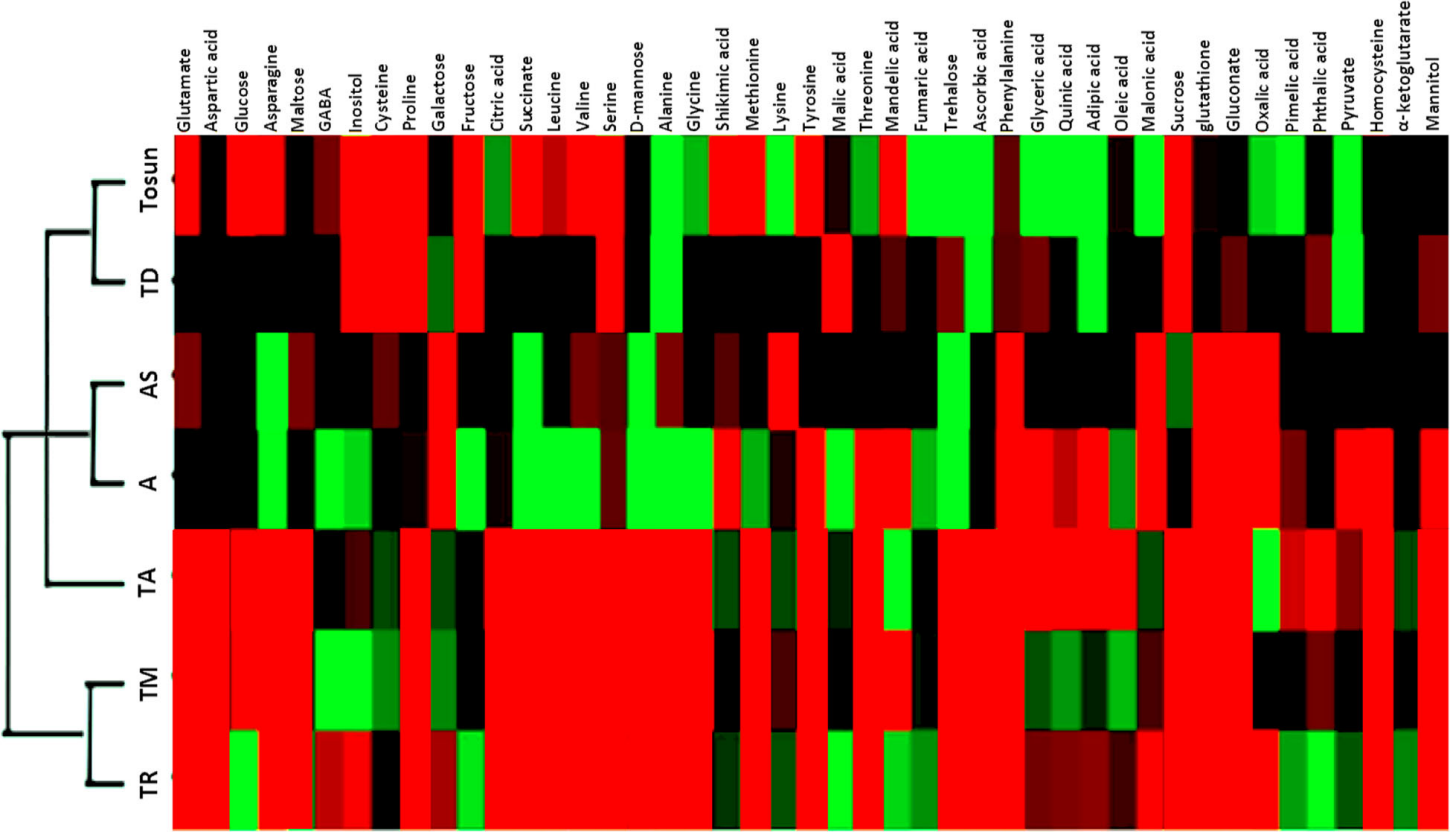
Hierarchical cluster analysis (HCA) revealed the differentially accumulated metabolites in seven Triticeae species after exposure to the drought stress (30% FC). The color scale red refers to high accumulation; black refers to moderate/normal accumulation and green refers to low accumulation. TR: TR39477 (*Triticum turgidum ssp. dicoccoides*), TM: IG132864 (*Triticum monococcum ssp. monococcum*), TA: Bolal (*Triticum aestivum ssp. aestivum*), A: Meyeri (*Aegilops tauschii ssp. tauschii*), AS: Ligustica (*Aegilops speltoides ssp. speltoids*), TD: TTD-22 (*Triticum turgidum ssp. dicoccoides*) and Tosun: Tosunbey (*Triticum aestivum ssp. aestivum*)

Hierarchical cluster analysis (HCA), on the other side, was performed to reveal the accumulation patterns of the metabolites. Figure 3 shows the accumulation patterns of 45 significantly altered metabolites after the exposure of plants to 30% FC for 16 days, analysed by HCA. On the basis of metabolite accumulation pattern, HCA presented two main clusters from all samples exposed to the drought stress. The smaller cluster consisted of two genotypes TR39477 and IG132864; Bolal placed next to them whereas the remaining four genotypes Meyeri, Ligustica, TTD-22 and Tosun together formed a bigger cluster as a result of their similar metabolite accumulation patterns.

### Identification of corresponding biological pathways and potential gene locations

**Fig. 4:**
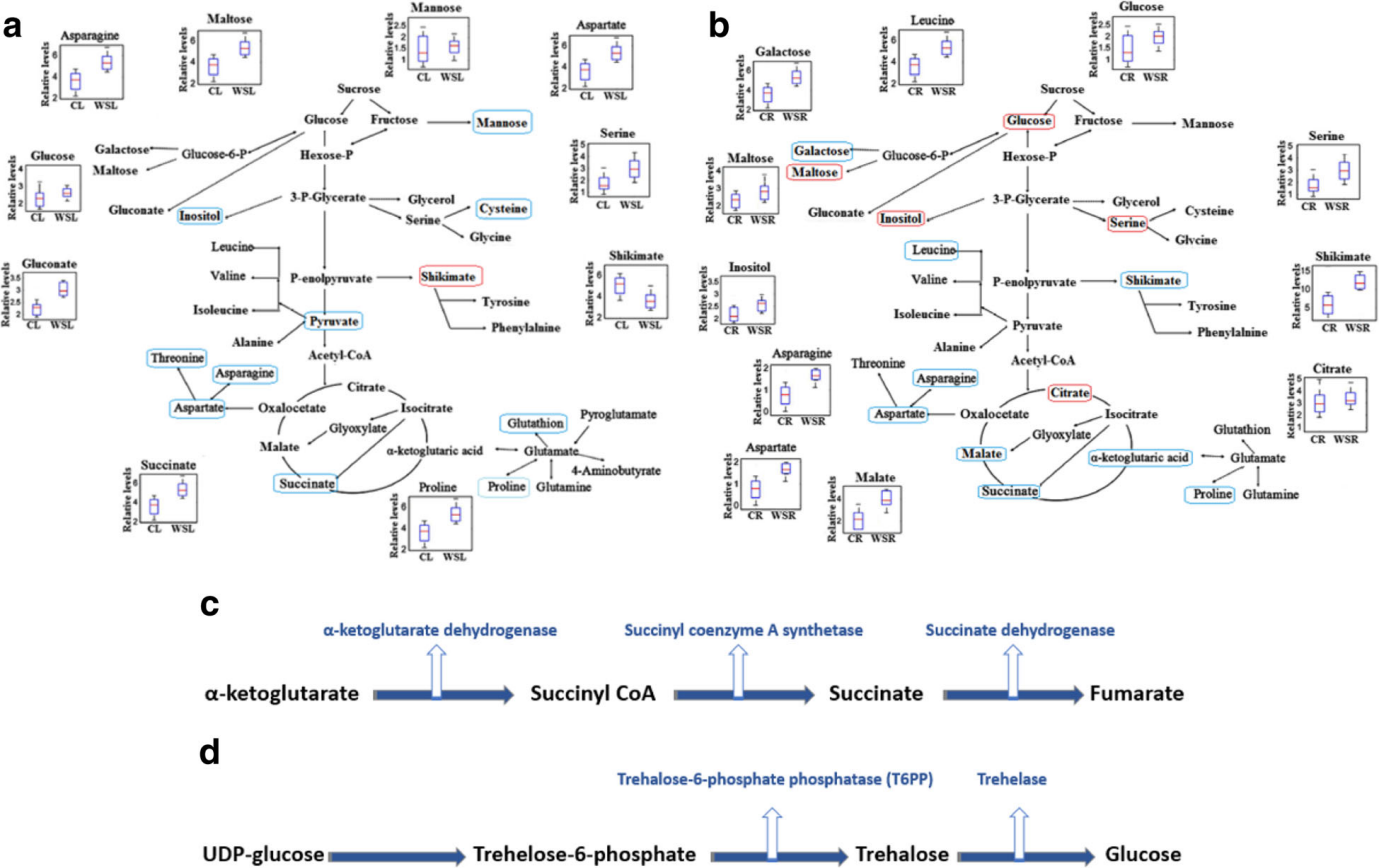
Leaf (**a**) and root (**b**) metabolites involved in the primary pathways in of Triticeae and its wild relatives under the drought stress. The significant (*P* < 0.05) and highly significant (*P* < 0.01) up-regulated metabolites were indicated in red and blue circles, respectively. Biochemical pathway for succinate (**c**) and trehelose synthesis (**d**)

All the metabolites affected by the drought stress were mapped to the biological pathways involved in the KEGG online database, which was assigned to 12 pathways in either treatment (Additional file 1: Table S4). The results showed that three pathways were enriched with the affected metabolites, as a consequence of the water stress. Furthermore, a metabolite-to-metabolite interaction network was constructed using all the altered metabolites as inputs that comprised metabolites for the drought stress exposure in wheat and its wild relatives. The biochemical pathways presenting the metabolites accumulated at high levels in the leaf and root samples of Triticeae species under the drought stress were shown in Fig. 4. Three metabolites including succinate, aspartate and trehalose were selected for further genome analysis due to their dramatically increased levels in TR39477, IG132864, and Bolal upon the drought stress treatment as well as their major role in energy producing biochemical pathway, known as TCA cycle.

Succinate is controlled by a relatively simple biochemical pathway involving three enzymatic steps as illustrated in Fig. 4c. Alpha-ketoglutarate is a substrate for conversion to succinyl-CoA by α-ketoglutarate dehydrogenase enzyme. succinyl-CoA is used to synthesise succinate through succinyl-CoA synthetase activity [23].

Potential wheat genes encoding the two enzymes were searched in WGSS and root transcriptome data [21]. FL-cDNA sequences with E-values less than 3e^−106^ were identified, including three with homology to α-ketoglutarate dehydrogenase and four with significant homology to succinyl-CoA. Analysis of the draft wheat genome sequence using wheat FL-cDNA as query sequence identified three copies of α-ketoglutarate dehydrogenase and four copies of succinyl-CoA related genes on the long arm of homologous chromosomes 1, 3 and 5 as presented in Table 3. The expected decrease in succinate level was observed in the wheat genotype TTD-22 that might be suppressing the succinyl CoA-related sequences under the drought stress. Expression of the genes in RNA-sequence data of our group [22] was also inspected where succinyl CoA-related genes showed a significant drop in the expression level in TTD-22. On the other hand, most genes in this pathway exhibited more gradual, yet significant, increased expression levels in TR39477.

**Table 3:** Selected three metabolites, their corresponding gene annotations and chromose locations on wheat genom

Gene ID	Annotated FL-cDNA	Gene annotation	Compound name	Wheat FL-cDNA	Wheat chromosome locations
15223122	D83378	Aspartate transaminase	Aspartate	AK333183	1AL, 1BL, 1DL
				AY621539	5AL, 5BL, 5DL
				AK334107	5AL, 5BL, 5DL
				BT009245	5AL, 5BL, 5DL
				BT009049	3AS, 5BS, 3DS
15229223	AK331389	α-ketoglutarate dehydrogenase	Succinate (Succinic acid)	AK330986	1AL, 3BL, 5DL
15228368	AK331389	Succinyl CoA synthetase		BT009368	1AS, 5AL, 6BL, 3DL
15227257	AK103775	Trehalose-6-phosphate synthase	Trehalose	FJ167677	1AL, 1BL, 1DL, 5DL
				AK331389	1AL, 1BL, 1DL, 5DL
				FJ167677	1AL, 1BL, 1DL, 5DL
				AK331389	1AL, 1BL, 1DL, 5DL
				FJ167677	1AL, 1BL, 1DL, 5DL
				AK331389	1AL, 1BL, 1DL, 5DL
				FJ167677	1AL, 1BL, 1DL, 5BL
				AK331389	1AL, 1BL, 1DL, 5BL
22330456	AK072132	Trehalose-6-phosphate phosphatase		AK333853	1AL, 1BL, 1DL, 3AL, 3BL, 3DL
				AK334843	1AL, 1BL, 1DL, 5AS, 5BS, 5BL
				FN564426	1AL, 1BL, 1DL, 5AS, 5BS, 5BL
				AK332212	1AL, 1DL, 3AL, 3AL, 3BL, 3DL
				AK331757	1AL, 1BL, 1DL
				BT009244	6AL, 6BL, 6DL
22331857	AK108163	Trehalase		AK331310	1AL, 1BL, 1DL

FL-cDNA sequences with E-values less than 3e-106 were identified

The other two most important drought stress specific metabolites selected were aspartate and trehalose. During the drought stress, aspartate transaminase enzyme was found to be responsible for the biosynthesis of aspartate from glutamate. Our previous studies indicated that aspartate transaminase belongs to a multi-gene family of which different homologous chromosomes (1, 3 and 5) contained almost six copies of these genes, instead of each copy present on 3AS and 3DS [2, 4, 22]. A general biochemical pathway having three enzymatic steps controls the accumulation of trehalose and uridine diphosphate glucose (UDP-glucose). Glucose-6-phosphate acts as a substrate for the conversion to trehalose through the trehalose-6-phosphate phosphatase (T6PP) enzyme. Finally, trehalase enzyme converts trehalose molecule into the glucose, as illustrated in Fig. 4d. The putative wheat genes encoding all enzymes involved in both biochemical pathways were identified in the WGSS. For comparative purposes, the identification of wheat cDNAs encoding aspartate transaminase, T6PP, T6PS and trehalase were performed. Analysis of the draft wheat genome sequence revealed different copy numbers of an enzyme related gene mentioned above on the long and short arms of various chromosomes of TR39477 and other drought stress tolerant genotypes. Of the TTD-22 and Tosunbey that lacked the drought stress-related sequences showed the expected decrease in metabolite levels [2, 4, 22].

## Discussion

The development of drought-tolerant crops seems a promising solution to increase the wheat crop yield under water-limited conditions, mainly to fulfil the staple food requirement for increasing world population [3, 8, 9, 24]. In this study, a broad spectrum of morphological and metabolic responses from seven Triticeae species was investigated under control and drought stress conditions. Significant morphological parameters measured were root length, surface area, average root diameter and overall root development. PCA, PLS-DA and HCA analyses were performed to determine the potential common metabolites of the drought stress mechanism in seven different Triticeae species. The metabolites showing significant accumulation under the drought stress were considered key metabolites and correlated with potential biochemical pathways, enzymes and gene locations for a better understanding of the stress mechanisms.

Several studies with different plants reported the inhibition of lateral roots and elongation of the root length, possibly to achieve a better water uptake from the deeper regions of the soil [25]. The enhanced vertical growth of the root under limited water conditions was found advantageous for crop productivity [26]. For instance, *Arabidopsis thaliana* root hairs became short and swollen in response to the water deficiency [25, 27], whereas the presence of very short and hairless root development under drought stress was reported in soil-grown *A. thaliana* [28]. In agreement with the literature, TR39477, IG132864, and Bolal also represented elongated root lengths under the drought stress condition while keeping their surface area large to absorb and store water.

Determination of the significantly altered metabolites accumulated upon drought stress was achieved with a non-targeted metabolite profiling analysis in Triticeae species using GC-MS technique. The most significant changes were observed in metabolites in the form of amino acid, organic acid, and sugars. TR39477, IG132864, and Bolal were found to be mainly accumulating proline, trehalose, glycine and some other amino acids upon drought stress treatment by 30% FC, considered as the potential drought stress-specific markers and osmoprotectants. The increased accumulation of these metabolites was previously reported for different plant species in which these metabolites were found to be responsible for drought stress tolerance [29–37]. Proline accumulation functions as an electron sink mechanism that can reduce the amount of singlet oxygen present, which causes lipid peroxidation of thylakoid membranes, providing evidence that it is a significant contributor to cellular redox balance [37–40].

The branch chain amino acids such leucine, valine, alanine also increased significantly in TR39477, IG132864 and Bolal samples unlike to the other genotypes under drought stress exposure. The increased accumulation of these branch chain amino acids was also reported in previous Arabidopsis studies [11, 41–43]. Less and Galili also indicated that the catabolic enzymes of amino acids increased rapidly in response to drought stress [44]. On the other hand, sugar and its derivatives such as galactose, mannose, fructose, mannitol and other non-reducing sugars and oligosaccharides provide a hydration shell around proteins under drought stress [45]. The increase of these sugars may provide an initial defensive state against further water loss.

Succinic acid or succinate is the primary intermediate component of ATP pathway, the citric acid cycle (Krebs cycle), which plays a vital role in energy production and involves in the regulation of mitochondrial TCA cycle [46]. The overproduction of NADH under drought stress inhibits all dehydrogenases (pyruvate dehydrogenase, isocitrate dehydrogenase, α-ketoglutarate dehydrogenase and citrate synthase) except the succinate dehydrogenase in TCA cycle which converts succinyl-CoA to succinate [47]. By over synthesis of succinate, mitochondria stores more ATP for unfavourable conditions [48]. The elevated level of succinate found in TR39477, which was previously characterised by its high tolerance against drought stress [1, 4, 16, 22], could be correlated with the efficient use of TCA cycle to produce more energy under water-limited conditions.

Succinic acid, trehalose and aspartic acid (aspartate) were selected for further genome analysis due to their potential involvement in biochemical pathways that were linked to the drought stress-specific responses [49, 50]. The primary focus for genomic analysis was drought stress tolerant genotypes, most specifically TR39477 due to the increased level of succinic acid. The results of genome analyses demonstrated that droughts stress tolerant wheat genotypes could be creditable for endorsing gene-to-metabolite networks. Therefore, the alteration in metabolic levels in sensitive and drought stress tolerant genotypes under control and drought stress conditions can be attributed to gene suppression or overexpression of the related chromosome arms.

Of the wheat genotypes that lack the succinyl CoA-related sequences, TTD-22 and Tosunbey were among the wheat genotypes that showed a decrease in the level of metabolites. These findings suggest that succinyl-CoA synthetase on 1AS, 5AL, 6BL, and 3DL might be a rate-limiting step in the succinate accumulation. However, the near to absent succinate level did not show a similar effect in Tosunbey, Ligustica and Meyeri, indicating that succinyl-CoA synthetase genes might be playing a different role in these species rather than succinate biosynthesis. A three-fold increase in the accumulation of succinate in TR39477 indicated that some unknown genes from primary biochemical pathways were regulating the accumulation of succinate in wheat. The metabolism of trehalose accumulation was controlled by post-translational modification pathways and regulatory networks [51]. Therefore, it was suggested that pathways specific genes might be located on 1AL, 1BL 1DL, 3BL, 3DL, 5AS, 6AL, 6BL and 6DL which were involved in the up-regulation of trehalose in TR39477. As discussed previously in the literature, the proteomic [4] and transcriptomic [22, 52] analyses of these cultivars provided candidate genes for the genetic manipulation of wheat cultivars in order to enhance drought stress tolerance, and the current metabolite data further validates these results.

## Conclusion

Drought stress affects the structure of plant cells and tissues. Hence a comprehensive omics approache (genomics, transcriptomics, proteomics, and metabolomics) will enhance our understanding of the underlying mechanisms of water deficiency in Triticeae, which will in turn help breeders to identify the responsive genes, proteins, metabolites for drought stress tolerance. This study indicated that drought stress treated leaves and roots of wheat and its wild genotypes have distinct mechanisms of metabolite accumulation and regulation, which is valuable for the better understanding of overall abiotic stress tolerance mechanisms. Triticeae species with high crop yields under the drought stress are expected to be developed in the future through the genetic transformation of novel genes identified in large-scale studies including metabolomics research.

## Additional file

Additional file 1: Figure S1. Root morphology of a normal and drought-stressed *Triticum aestivum* (Bolal). Normal conditions (a), drought stress condition (b), light Microscopy (10X) images of lateral root length (c) and diameter (d), normal primary root diameter of 19.17 µm (e), drought stress primary & secondary root diameters of 13.8 µm (f). **Figure S2.** GC-MS spectra for a typical control leave sample (lower pannel) and drought-treated leave sample (upper pannel). *Aegilops speltoides* (A) *Triticum dicoccoides* (TR39477) (B) *Triticum dicoccoides* (TTD-22) (C) and *Triticum aestivum* (Bolal) (D). **Figure S3.** GC-MS spectra for a typical control leave sample (lower pannel) and drought-treated leave sample (upper pannel). *Triticum aestivum* (Tosunbey) (E), *Triticum monococcum* (F) and *Aegilops tauschii* (G). Complete chromatographic time was 5.0-40.0 min. **Figure S4.** GC-MS spectra for a typical control root sample (lower pannel) and drought-treated root sample (upper pannel). *Aegilops speltoides* (A), *Triticum dicoccoides* (TR39477) (B), *Triticum dicoccoides* (TTD-22) (C) and *Triticum aestivum* (Bolal) (D). **Figure S5.** GC-MS spectra for a typical control root sample (lower pannel) and drought-treated root sample (upper pannel). *Triticum aestivum* (Tosunbey) (E), *Triticum monococcum* (F) and *Aegilops tauschii* (G). **Table S1.** Stress responsive metabolites identified in leaf samples. Leaf metabolites, the fold changesx in the concentrations of each metabolite between control (CL) and drought-stressed (DSL) groups using the formula log2(Drought treated/Control) and variable importance in the projection (VIP) of the typical/representative sample (TR39477). **Table S2.** Water-stress responsive metabolites identified in root. Root metabolites, the fold changesx in the concentrations of each metabolite between control (CR) and drought-stressed (DSR) groups using the formula log2(Drought treated/Control) and variable importance in the projection (VIP) of the typical/representative sample (TR39477). **Table S3.** Principal Component Analysis (PCA) and partial least-squares-discriminant analysis (PLS-DA) results. The explanation and predictability values measured for the first two Principal Components (PCs) were found 71.2% and 42.6%, respectively. **Table S4.** The KEGG pathways (R-software) of the altered metabolites exposure to drought stress in wheat leaves and root samples. (DOCX 1930 kb)

## Abbreviations

CL: Control leaves
CR: Control roots
DSL: Drought stress treated leaves
DSR: Drought stress treated roots
FC: Field capacity
FL-cDNA: Full-length complementary DNA
GC-MS: Gas chromatography-mass spectrometry
PCA: Principal component analysis
PCs: Principal components
PLS-DA: Partial least-squares-discriminant analysis
QTL: Quantitative trait locus
RL: Root length
SA: Surface area
T6PP: Trehalose-6-phosphate phosphatase
T6PS: Trehelose-6-phosphate synthase
TICs: Total ion chromatograms
UDP-glucose: Uridine diphosphate glucose
VIP: Variable importance in the projection
WGSS: Wheat genome survey sequences
