# Supplementary material for: Comparative metabolite profiling of drought stress in roots and leaves of seven Triticeae species
Source: BMC Genomics. 2017 Dec 15;18:969. doi: 10.1186/s12864-017-4321-2 (PMC5731210; doi:10.1186/s12864-017-4321-2)
Supplement: Additional file 1: Figure S1. — Root morphology of a normal and drought-stressed Triticum aestivum (Bolal). Normal conditions (a), drought stress condition (b), light Microscopy (10X) images of lateral root length (c) and diameter (d), normal primary root diameter of 19.17 µm (e), drought stress primary & secondary root diameters of 13.8 µm (f). Figure S2. GC-MS spectra for a typical control leave sample (lower pannel) and drought-treated leave sample (upper pannel). Aegilops speltoides (A) Triticum dicoccoides (TR39477) (B) Triticum dicoccoides (TTD-22) (C) and Triticum aestivum (Bolal) (D). Figure S3. GC-MS spectra for a typical control leave sample (lower pannel) and drought-treated leave sample (upper pannel). Triticum aestivum (Tosunbey) (E), Triticum monococcum (F) and Aegilops tauschii (G). Complete chromatographic time was 5.0-40.0 min. Figure S4. GC-MS spectra for a typical control root sample (lower pannel) and drought-treated root sample (upper pannel). Aegilops speltoides (A), Triticum dicoccoides (TR39477) (B), Triticum dicoccoides (TTD-22) (C) and Triticum aestivum (Bolal) (D). Figure S5. GC-MS spectra for a typical control root sample (lower pannel) and drought-treated root sample (upper pannel). Triticum aestivum (Tosunbey) (E), Triticum monococcum (F) and Aegilops tauschii (G). Table S1. Stress responsive metabolites identified in leaf samples. Leaf metabolites, the fold changesx in the concentrations of each metabolite between control (CL) and drought-stressed (DSL) groups using the formula log2(Drought treated/Control) and variable importance in the projection (VIP) of the typical/representative sample (TR39477). Table S2. Water-stress responsive metabolites identified in root. Root metabolites, the fold changesx in the concentrations of each metabolite between control (CR) and drought-stressed (DSR) groups using the formula log2(Drought treated/Control) and variable importance in the projection (VIP) of the typical/representative sample (TR39477). Table S3. Principal Component [file 12864_2017_4321_MOESM1_ESM.docx]

**Comparative metabolite profiling of drought stress in roots and leaves of seven Triticeae species**

Naimat Ullah **^1^**, Meral Yüce **^2^**, Z. Neslihan Öztürk Gökçe **^3^**, and Hikmet Budak **^4*^**

^1^ Sabanci University, Faculty of Engineering and Natural Sciences, 34956, Istanbul, Turkey.

^2^ Sabanci University, Nanotechnology Research and Application Centre, 34956, Istanbul, Turkey.

^3^ Nigde Omer Halisdemir University, Ayhan Sahenk Faculty of Agricultural Sciences and Technologies, 51240, Nigde, Turkey.

^4^ Montana State University, Department of Plant Science and Plant Pathology, Bozeman, MT, USA.

***Corresponding author address:** Prof Dr. Hikmet Budak, Montana State University, Department of Plant Science and Plant Pathology, Bozeman, MT, USA.

**E-mail:** [hikmet.budak@montana.edu](mailto:hikmet.budak@montana.edu)

**Office:** +1-406-994-6717

**Fax:** +1-406-994-1848

# GC-MS Spectra data

1. Sucrose

2. Trehalose

3. Mannitol

4. Maltose

5. Proline

6. Glutamate

7. Alanine

8. Lycine

9. Asparagines

10. Methionine

11. Threonine

12. Phenylalanine

13. Homocysteine

14. Serine

15. Valine

16. Tyrosine

17. Succinate

18. Citrate

19. Aspartate

20. Gluconate

21. Glutathione

*“*” and “**” indicate the significance (P<0.05) and highly significance (P<0.01) level. Metabolites were extracted from the leaf and root tissue samples in triplicates from all seven Triticeae species for each of the four experimental groups, including drought stress treated leaves (DSL), drought stress treated roots (DSR), control leaves (CL) and control roots (CR).*

# Figure Captions

[**Figure 1.** Root morphology of a normal and drought-stressed Triticum aestivum (Bolal). Normal conditions **(a)**, drought stress condition **(b)**, light Microscopy (10X) images of lateral root length **(c)** and diameter **(d)**, normal primary root diameter of 19.17 µm **(e)**, drought stress primary & secondary root diameters of 13.8 µm **(f)**. 5](#_Toc499286405)

[**Figure 2.** GC-MS spectra for a typical control leave sample (lower pannel) and drought-treated leave sample (upper pannel). Aegilops speltoides **(A)** Triticum dicoccoides (TR39477) **(B)** Triticum dicoccoides (TTD-22) **(C)** and Triticum aestivum (Bolal) **(D)**. 6](#_Toc499286406)

[**Figure 3**. GC-MS spectra for a typical control leave sample (lower pannel) and drought-treated leave sample (upper pannel). Triticum aestivum (Tosunbey) **(E),** Triticum monococcum **(F)** and Aegilops tauschii **(G)**. Complete chromatographic time was 5.0-40.0 min. 7](#_Toc499286407)

[**Figure 4.** GC-MS spectra for a typical control root sample (lower pannel) and drought-treated root sample (upper pannel). Aegilops speltoides **(A),** Triticum dicoccoides (TR39477) **(B),** Triticum dicoccoides (TTD-22) **(C)** and Triticum aestivum (Bolal) **(D)**. 8](#_Toc499286408)

[**Figure 5.** GC-MS spectra for a typical control root sample (lower pannel) and drought-treated root sample (upper pannel). Triticum aestivum (Tosunbey) **(E),** Triticum monococcum **(F)** and Aegilops tauschii **(G)**. 9](#_Toc499286409)

# Table Captions

**[Table 1.](#_Toc499286442)** [Stress responsive metabolites identified in leaf samples. Leaf metabolites, the fold changes](#_Toc499286442)^[x](#_Toc499286442)^ [in the concentrations of each metabolite between control (CL) and drought-stressed (DSL) groups using the formula log](#_Toc499286442)_[2](#_Toc499286442)_[(Drought treated/Control) and variable importance in the projection (VIP) of the typical/representative sample (TR39477). 10](#_Toc499286442)

[**Table 2.** Water-stress responsive metabolites identified in root. Root metabolites, the fold changesx in the concentrations of each metabolite between control (CR) and drought-stressed (DSR) groups using the formula log_2_(Drought treated/Control) and variable importance in the projection (VIP) of the typical/representative sample (TR39477). 12](#_Toc499286443)

[**Table 3.** Principal Component Analysis (PCA) and partial least-squares-discriminant analysis (PLS-DA) results. The explanation and predictability values measured for the first two Principal Components (PCs) were found 71.2% and 42.6%, respectively. 14](#_Toc499286444)

[**Table 4.** The KEGG pathways (R-software) of the altered metabolites exposure to drought stress in wheat leaves and root samples. 14](#_Toc499286445)


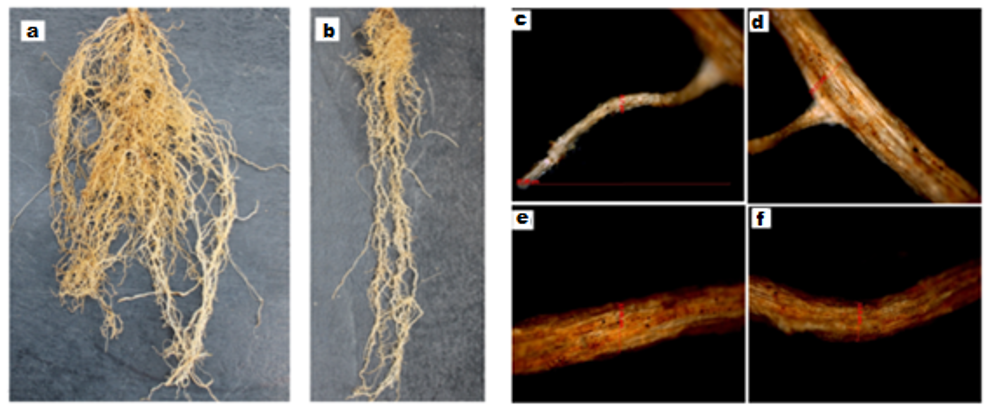


**Figure 1.** Root morphology of a normal and drought-stressed Triticum aestivum (Bolal). Normal conditions **(a)**, drought stress condition **(b)**, light Microscopy (10X) images of lateral root length **(c)** and diameter **(d)**, normal primary root diameter of 19.17 µm **(e)**, drought stress primary & secondary root diameters of 13.8 µm **(f)**.


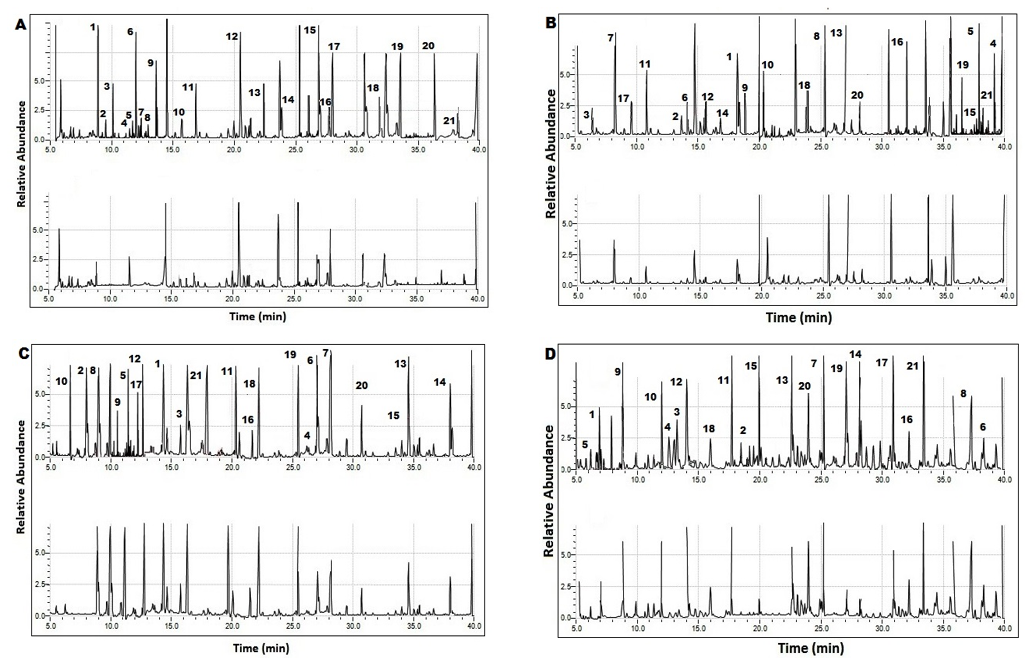


**Figure 2.** GC-MS spectra for a typical control leave sample (lower pannel) and drought-treated leave sample (upper pannel). Aegilops speltoides **(A)** Triticum dicoccoides (TR39477) **(B)** Triticum dicoccoides (TTD-22) **(C)** and Triticum aestivum (Bolal) **(D)**.


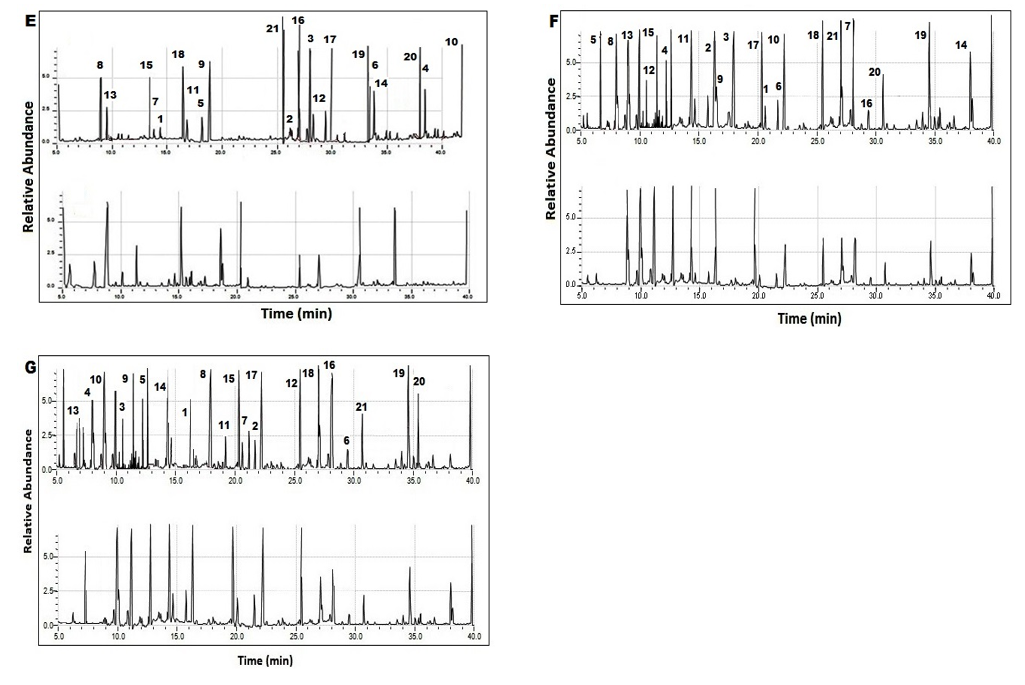


**Figure 3**. GC-MS spectra for a typical control leave sample (lower pannel) and drought-treated leave sample (upper pannel). Triticum aestivum (Tosunbey) **(E),** Triticum monococcum **(F)** and Aegilops tauschii **(G)**. Complete chromatographic time was 5.0-40.0 min.


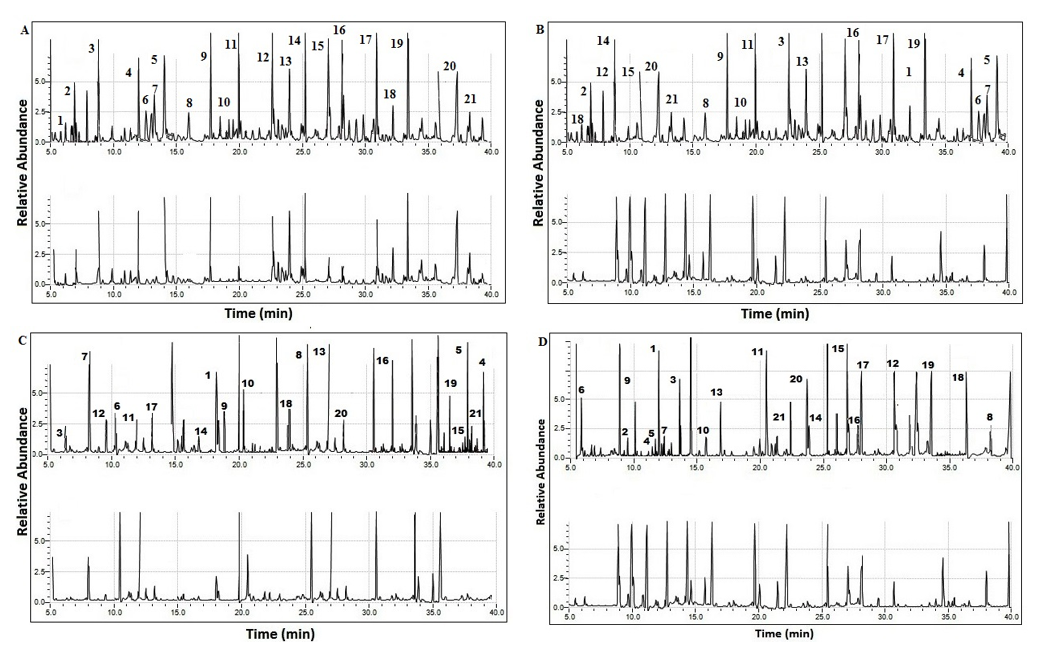


**Figure 4.** GC-MS spectra for a typical control root sample (lower pannel) and drought-treated root sample (upper pannel). Aegilops speltoides **(A),** Triticum dicoccoides (TR39477) **(B),** Triticum dicoccoides (TTD-22) **(C)** and Triticum aestivum (Bolal) **(D)**.


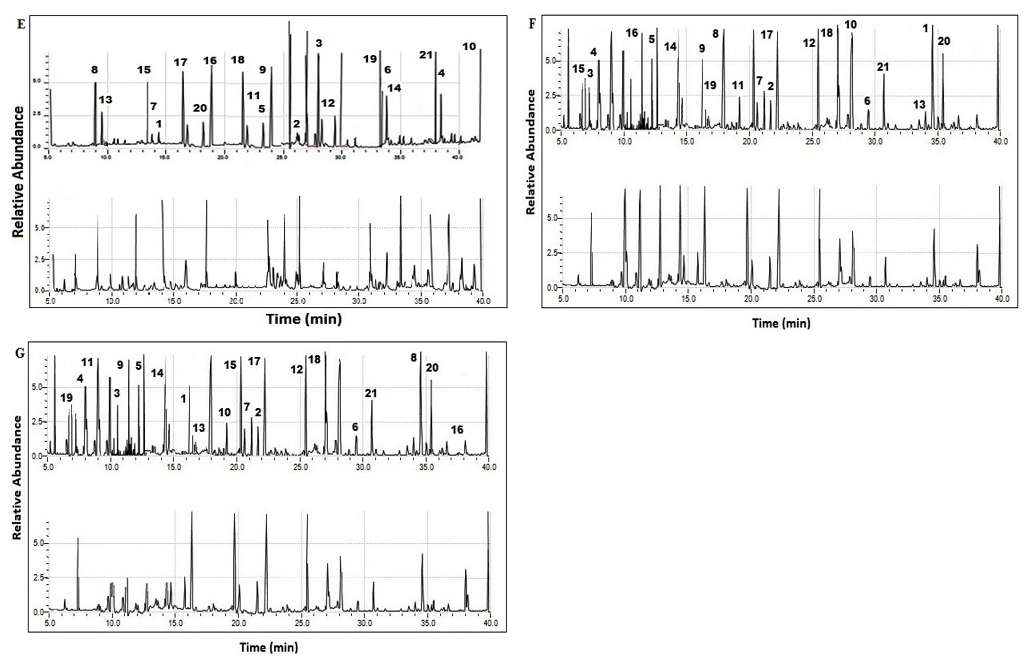


**Figure 5.** GC-MS spectra for a typical control root sample (lower pannel) and drought-treated root sample (upper pannel). Triticum aestivum (Tosunbey) **(E),** Triticum monococcum **(F)** and Aegilops tauschii **(G)**.

**Table 1.** Stress responsive metabolites identified in leaf samples. Leaf metabolites, the fold changes^x^ in the concentrations of each metabolite between control (CL) and drought-stressed (DSL) groups using the formula log_2_(Drought treated/Control) and variable importance in the projection (VIP) of the typical/representative sample (TR39477).

| **m/z** | **RT (min)** | **SI (%)** | **Metabolite** | **DSL-Average** | **CL-average** | **SDE.DSL** | **SDE.CL** | **VIP** | **Fold change** | **T-test** |
| --- | --- | --- | --- | --- | --- | --- | --- | --- | --- | --- |
| 132.0205 | 5,499 | 96 | Sucrose | 251431.2845 | 390449.1170 | 103547.2656 | 51986.2213 | 1.752 | 2.5124 | 0.0069 |
| 101.7112 | 6,234 | 89 | Trehalose | 25964.1344 | 90523.4619 | 70466.3138 | 22455.3902 | 1.359 | 3.5821 | 0.0018 |
| 116.0193 | 6,776 | 99 | Glucose | 5591221.797 | 10130466.42 | 5045293.597 | 5045293.597 | 0.5704 | 0.9179 | 0.0011 |
| 219.9998 | 8,599 | 93 | Maltose | 409006.3770 | 541189.2128 | 49252.4174 | 49252.4174 | 1.5704 | 1.9059 | 0.0011 |
| 255.9982 | 9,572 | 95 | Proline | 66222.9467 | 113220.9117 | 39403.2342 | 17961.5473 | 1.321 | 3.9542 | 0.0026 |
| 66.0865 | 10,433 | 92 | Glutamate | 104787.8757 | 165384.8123 | 60649.2055 | 35147.9899 | 1.1819 | 1.1574 | 0.0374 |
| 132.9994 | 11,688 | 99 | Malonic acid | 2622465.079 | 3239226.214 | 227192.2793 | 227192.2793 | 0.2131 | 2.3454 | 0.0256 |
| 73.0719 | 13,533 | 94 | Glycine | 15308773.50 | 21576528.21 | 5291598.252 | 1821437.269 | 1.4334 | 1.3621 | 0.0008 |
| 219.0470 | 14,397 | 95 | Asparagine | 272193.0932 | 428909.0846 | 141886.4577 | 79207.2451 | 1.2545 | 1.3501 | 0.0027 |
| 358.9890 | 15,033 | 96 | Methionine | 181826.9050 | 309026.2336 | 102279.5146 | 74211.3542 | 1.2879 | 2.1841 | 0.0176 |
| 393.9973 | 15,642 | 98 | Oleic acid | 116696.7910 | 247305.4145 | 117373.1155 | 75224.5114 | 0.5409 | -1.1414 | 0.0195 |
| 372.9999 | 16,085 | 99 | Ascorbic acid | 892584.5688 | 1727014.140 | 383612.8697 | 398008.6959 | 0.5939 | -1.0944 | 0.0015 |
| 433.9814 | 17,066 | 98 | Homocysteine | 23664.3172 | 36203.5948 | 10026.7400 | 7955.5609 | 1.2974 | 1.3052 | 0.0199 |
| 465.0958 | 18,061 | 99 | Serine | 99669.7410 | 17378.6878 | 15333.5729 | 9805.3277 | 1.3457 | 0.9621 | 0.0069 |
| 307.1537 | 18,756 | 97 | Lysine | 22227193.82 | 14610088.56 | 6621050.428 | 1041945.072 | 0.7221 | -2.1965 | 0.0001 |
| 87.0059 | 19,967 | 91 | Leucine | 536858.6475 | 356788.9754 | 90358.2018 | 60873.7700 | 0.4523 | -2.6475 | 0.0161 |
| 319.1864 | 20,393 | 99 | Succinate | 43354641.49 | 36692643.80 | 4508559.663 | 100392.6735 | 1.2116 | 1.2954 | 0.0338 |
| 424.0809 | 21,032 | 89 | Phthalic acid | 5382.4716 | 1205.0130 | 821.7256 | 821.7256 | 0.2974 | 1.3052 | 0.0199 |
| 319.1101 | 21,413 | 88 | Aspartate | 2304711.319 | 3300932.350 | 281755.353 | 281755.353 | 1.548 | 2.1965 | 0.0001 |
| 337.1527 | 22,926 | 98 | Mannose | 59060.7969 | 40803.9515 | 11314.0262 | 14261.8330 | 0.4334 | 1.3621 | 0.0108 |
| 79.0102 | 23,467 | 99 | Glyceric acid | 625910.6571 | 477717.6815 | 85894.0185 | 116963.662 | 0.2545 | 1.3501 | 0.0227 |
| 132.0205 | 24,940 | 96 | Threonine | 251431.2845 | 390449.1170 | 103547.2656 | 51986.2213 | 1.2131 | 2.3454 | 0.0056 |
| 101.7112 | 26,330 | 95 | Phenylalanine | 25964.1344 | 90523.4619 | 70466.3138 | 22455.3902 | 1.821 | 2.1414 | 0.0012 |
| 116.0193 | 27,053 | 98 | α-ketogluteric acid | 5591221.797 | 10130466.42 | 5045293.597 | 5045293.59 | 0.263 | -0.835 | 0.0453 |
| 219.9998 | 28,165 | 95 | Inositol | 409006.3770 | 541189.2128 | 49252.4174 | 49252.4174 | 0.321 | 0.9542 | 0.0126 |
| 255.9982 | 28,929 | 96 | Galactose | 66222.9467 | 113220.9117 | 39403.2342 | 17961.5473 | 0.1819 | 1.1574 | 0.0374 |
| 66.0865 | 29,997 | 98 | Fructose | 104787.8757 | 165384.8123 | 60649.2055 | 35147.9899 | 0.3418 | 1.3114 | 0.0077 |
| 132.9994 | 31,367 | 99 | Alanine | 2622465.079 | 3239226.214 | 227192.2793 | 227192.279 | 1.3418 | 1.3114 | 0.0077 |
| 73.0719 | 32,270 | 98 | Tyrosine | 15308773.50 | 21576528.21 | 5291598.252 | 1821437.26 | 1.5939 | 1.0944 | 0.0015 |
| 219.0470 | 32,867 | 99 | Quinic acid | 272193.0932 | 428909.0846 | 141886.4577 | 79207.2451 | 0.2879 | 2.1841 | 0.0176 |
| 358.9890 | 33,300 | 97 | Citrate (Citric acid) | 181826.9050 | 309026.2336 | 102279.5146 | 74211.3542 | 1.6205 | 1.6365 | 0.0003 |
| 393.9973 | 33,430 | 98 | Oxalic acid | 116696.7910 | 247305.4145 | 117373.1155 | 75224.5114 | 0.5243 | 2.1414 | 0.0012 |
| 372.9999 | 33,626 | 95 | Gluconate | 892584.5688 | 1727014.140 | 383612.8697 | 398008.695 | 1.4523 | 2.6475 | 0.0161 |
| 433.9814 | 34,689 | 89 | Pimelic acid | 23664.3172 | 36203.5948 | 10026.7400 | 7955.5609 | 0.3457 | -0.9621 | 0.0069 |
| 465.0958 | 35,012 | 99 | Mannitol | 99669.7410 | 17378.6878 | 15333.5729 | 9805.3277 | 1.263 | 1.835 | 0.0453 |
| 307.1537 | 35,233 | 93 | Fumeric acid | 22227193.82 | 14610088.56 | 6621050.428 | 1041945.07 | 0.2116 | -1.2954 | 0.0338 |
| 87.0059 | 35,427 | 95 | Mandelic acid | 536858.6475 | 356788.9754 | 90358.2018 | 60873.7700 | 0.6205 | -1.6365 | 0.0003 |
| 319.1864 | 36,069 | 92 | Valine | 43354641.49 | 36692643.80 | 4508559.663 | 100392.6735 | 1.5409 | 1.1414 | 0.0095 |
| 424.0809 | 36,299 | 99 | Cysteine | 5382.4716 | 1205.0130 | 821.7256 | 821.7256 | 0.3901 | -1.3912 | 0.0144 |
| 319.1101 | 36,435 | 94 | Shikimic acid | 2304711.319 | 3300932.350 | 281755.3536 | 281755.3536 | 0.7221 | -2.1965 | 0.0001 |
| 337.1527 | 38,628 | 97 | Glutathione | 59060.7969 | 40803.9515 | 11314.0262 | 14261.8330 | 1.3901 | 1.3912 | 0.0044 |
| 79.0102 | 38,867 | 92 | ɣ-Aminobutyric acid (GABA) | 625910.6571 | 477717.6815 | 85894.0185 | 116963.6621 | 0.2767 | -0.5124 | 0.0069 |
| 319.1101 | 39,064 | 94 | Pyruvate | 181826.9050 | 309026.2336 | 102279.5146 | 74211.3542 | 0.1003 | -0.7251 | 0.0377 |
| 337.1527 | 39,471 | 81 | Malic acid | 116696.7910 | 247305.4145 | 117373.1155 | 75224.5114 | 0.4523 | -2.6475 | 0.0161 |
| 79.0102 | 39,793 | 83 | Adipic acid | 892584.5688 | 1727014.140 | 383612.8697 | 398008.6959 | 0.3901 | -1.3912 | 0.0144 |

**Table 2.** Water-stress responsive metabolites identified in root. Root metabolites, the fold changesx in the concentrations of each metabolite between control (CR) and drought-stressed (DSR) groups using the formula log_2_(Drought treated/Control) and variable importance in the projection (VIP) of the typical/representative sample (TR39477).

| **m/z** | **RT (min)** | **SI (%)** | **Metabolite** | **DSR-aveage** | **CR-aveage** | **SDE.DSR** | **SDE.CR** | **VIP** | **Fold change** | **T-test** |
| --- | --- | --- | --- | --- | --- | --- | --- | --- | --- | --- |
| 132.0205 | 5,504 | 96 | Sucrose | 132175.27 | 390449.12 | 127883.36 | 51986.22 | 1.10 | 3.51 | 0.01 |
| 101.7112 | 6,241 | 95 | Trehalose | 20693.48 | 90523.46 | 25373.72 | 22455.39 | 1.37 | 2.58 | 0.00 |
| 116.0193 | 8,854 | 94 | Glucose | 3968285.96 | 10130466.42 | 3710097.47 | 5045293.60 | 0.58 | -0.92 | 0.01 |
| 219.9998 | 10,140 | 95 | Maltose | 317053.95 | 541189.21 | 250371.68 | 49252.42 | 1.59 | 2.92 | 0.00 |
| 255.9982 | 11,360 | 96 | Proline | 43128.38 | 113220.91 | 29605.79 | 17961.55 | 1.33 | 3.95 | 0.02 |
| 114.0049 | 12,201 | 98 | Glutamate | 112089.62 | 243641.28 | 130356.50 | 35147.99 | 1.15 | 2.16 | 0.00 |
| 132.9994 | 14,200 | 99 | Malonic acid | 1816819.88 | 3239226.21 | 982936.81 | 227192.28 | 0.37 | -2.11 | 0.03 |
| 73.0719 | 15,187 | 98 | Glycine | 11263933.05 | 21576528.21 | 6131715.67 | 1821437.27 | 1.26 | 3.36 | 0.01 |
| 158.0967 | 16,093 | 99 | Asparagine | 675655.08 | 2175441.83 | 1018227.63 | 1608227.02 | 1.01 | 4.35 | 0.00 |
| 142.0977 | 18,610 | 97 | Methionine | 2762313.35 | 11664490.61 | 2695770.93 | 8309725.61 | 1.06 | 1.18 | 0.02 |
| 393.9973 | 20,392 | 96 | Oleic acid | 12241.74 | 247305.41 | 24404.78 | 117373.12 | 0.56 | 1.14 | 0.02 |
| 100.9987 | 21,000 | 95 | Ascorbic acid | 128052.78 | 445599.61 | 174469.43 | 377787.57 | 0.97 | 1.09 | 0.00 |
| 372.9999 | 21,407 | 96 | Homocysteine | 313390.60 | 1727014.14 | 277807.35 | 383612.87 | 1.02 | 3.31 | 0.02 |
| 152.9097 | 22,272 | 94 | Serine | 42275.50 | 69213.73 | 23054.00 | 20014.46 | 1.11 | 3.96 | 0.01 |
| 182.9957 | 24,518 | 93 | Lysine | 74527.96 | 156391.99 | 52675.11 | 76181.02 | 0.66 | 2.20 | 0.00 |
| 260.0053 | 25,451 | 98 | Leucine | 43756.75 | 107924.65 | 46282.75 | 67801.62 | 0.12 | 2.56 | 0.02 |
| 172.0850 | 26,136 | 93 | Succinate | 393737.40 | 786351.12 | 321374.31 | 342415.86 | 1.70 | 3.30 | 0.03 |
| 451.0000 | 26,903 | 92 | Phthalic acid | 132370.38 | 1181980.18 | 225439.02 | 994334.98 | 0.12 | -1.31 | 0.02 |
| 595.0824 | 27,193 | 91 | Aspartate (Aspartic acid) | 7462.27 | 16839.37 | 6936.30 | 9805.33 | 1.86 | 2.15 | 0.00 |
| 173.0187 | 27,698 | 99 | Mannose | 1268376.95 | 2779319.56 | 579978.06 | 1041945.07 | 0.90 | -1.36 | 0.01 |
| 227.9750 | 28,171 | 89 | Glyceric acid | 114902.08 | 183897.79 | 53732.92 | 60873.77 | 0.46 | -1.35 | 0.02 |
| 132.0205 | 28,629 | 88 | Threonine | 132175.27 | 390449.12 | 127883.36 | 51986.22 | 1.98 | 3.35 | 0.01 |
| 101.7112 | 29,179 | 98 | Phenylalanine | 20693.48 | 90523.46 | 25373.72 | 22455.39 | 1.40 | 2.15 | 0.00 |
| 116.0193 | 30,004 | 99 | α-ketogluteric acid | 3968285.96 | 10130466.42 | 3710097.47 | 5045293.60 | 0.85 | 0.84 | 0.05 |
| 219.9998 | 31,423 | 96 | Inositol | 317053.95 | 541189.21 | 250371.68 | 49252.42 | 0.74 | -0.95 | 0.00 |
| 255.9982 | 31,794 | 95 | Galactose | 43128.38 | 113220.91 | 29605.79 | 17961.55 | 0.32 | -1.16 | 0.04 |
| 114.0049 | 32,114 | 98 | Fructose | 112089.62 | 243641.28 | 130356.50 | 35147.99 | 0.65 | -1.23 | 0.01 |
| 132.9994 | 32,563 | 98 | Alanine | 1816819.88 | 3239226.21 | 982936.81 | 227192.28 | 1.75 | 2.31 | 0.01 |
| 73.0719 | 32,782 | 97 | Tyrosine | 11263933.05 | 21576528.21 | 6131715.67 | 1821437.27 | 1.95 | 3.09 | 0.00 |
| 158.0967 | 33,196 | 96 | Quinic acid | 675655.08 | 2175441.83 | 1018227.63 | 1608227.02 | 0.72 | -2.18 | 0.02 |
| 142.0977 | 33,433 | 88 | Citrate (Citric acid) | 2762313.35 | 11664490.61 | 2695770.93 | 8309725.61 | 1.97 | 1.64 | 0.00 |
| 393.9973 | 33,635 | 96 | Oxalic acid | 12241.74 | 247305.41 | 24404.78 | 117373.12 | 0.56 | -2.14 | 0.00 |
| 100.9987 | 34,009 | 95 | Gluconate | 128052.78 | 445599.61 | 174469.43 | 377787.57 | 1.45 | 3.65 | 0.01 |
| 372.9999 | 34,306 | 94 | Pimelic acid | 313390.60 | 1727014.14 | 277807.35 | 383612.87 | 0.01 | 0.13 | 0.01 |
| 152.9097 | 34,699 | 95 | Mannitol | 42275.50 | 69213.73 | 23054.00 | 20014.46 | 1.89 | 2.84 | 0.01 |
| 182.9957 | 34,959 | 96 | Fumeric acid | 74527.96 | 156391.99 | 52675.11 | 76181.02 | 0.75 | 1.26 | 0.03 |
| 260.0053 | 35,201 | 98 | Mandelic acid | 43756.75 | 107924.65 | 46282.75 | 67801.62 | 0.56 | 1.64 | 0.00 |
| 172.0850 | 35,567 | 99 | Valine | 393737.40 | 786351.12 | 321374.31 | 342415.86 | 1.89 | 3.14 | 0.00 |
| 451.0000 | 37,874 | 98 | Cysteine | 132370.38 | 1181980.18 | 225439.02 | 994334.98 | 0.34 | 1.65 | 0.02 |
| 595.0824 | 38,233 | 99 | Shikimic acid | 7462.27 | 16839.37 | 6936.30 | 9805.33 | 0.22 | 1.20 | 0.00 |
| 173.0187 | 38,368 | 97 | Glutathione | 1268376.95 | 2779319.56 | 579978.06 | 1041945.07 | 1.85 | 2.39 | 0.00 |
| 227.9750 | 38,542 | 96 | ɣ-Aminobutyric acid (GABA) | 114902.08 | 183897.79 | 53732.92 | 60873.77 | 0.56 | 0.75 | 0.00 |
| 595.0824 | 38,839 | 95 | Pyruvate | 7462.27 | 16839.37 | 6936.30 | 9805.33 | 0.19 | 0.34 | 0.03 |
| 173.0187 | 39,067 | 96 | Malic acid | 1268376.95 | 2779319.56 | 579978.06 | 1041945.07 | 0.70 | 1.89 | 0.02 |
| 227.9750 | 39,797 | 89 | Adipic acid | 114902.08 | 183897.79 | 53732.92 | 60873.77 | 0.23 | 2.85 | 0.01 |

**Table 3.** Principal Component Analysis (PCA) and partial least-squares-discriminant analysis (PLS-DA) results. The explanation and predictability values measured for the first two Principal Components (PCs) were found 71.2% and 42.6%, respectively.

|  |  | **CL-DSL-CR-DSR** | **CL-DSL** | **CR-DSR** | **DSL-DSR** | **CL-CR** |
| --- | --- | --- | --- | --- | --- | --- |
| **PCA** | **R^2^X** | 0.712 | 0.691 | 0.689 | 0.662 | 0.651 |
|  | **Q^2^** | 0.426 | 0.382 | 0.452 | 0.469 | 0.472 |
| **PLS-DA** | **R^2^X** | 0.522 | 0.412 | 0.685 | 0.656 | 0.694 |
|  | **R^2^Y** | 0.513 | 0.891 | 0.971 | 0.482 | 0.461 |
|  | **Q^2^** | 0.361 | 0.551 | 0.642 | 0.375 | 0.058 |

**Table 4.** The KEGG pathways (R-software) of the altered metabolites exposure to drought stress in wheat leaves and root samples.

| **Label** | **P-value** | **Adjusted p-value** | **Background_count** | **Count** | **Compounds** |
| --- | --- | --- | --- | --- | --- |
| **Galactose metabolism** | 0.00 | 0.00 | 8 | 3 | Glucose, galactose , maltose |
| **Starch and sucrose metabolism** | 0.00 | 0.00 | 6 | 2 | Sucrose, trehalose |
| **Citrate cycle (TCA cycle)** | 0.01 | 0.04 | 9 | 7 | Aspartate, mannose, serine, succinate, glutamate, proline, asparagine |
| **Pentose phosphate pathway** | 0.02 | 0.07 | 5 | 3 | Galactose, maltose, glucose-6-phosphate |
| **Glycine, serine and threonine metabolism** | 0.04 | 0.11 | 8 | 2 | Serine, threonine |
| **Cysteine and methionine metabolism** | 0.05 | 0.13 | 9 | 2 | Cysteine, methionine |
| **Biosynthesis of phenylpropanoids** | 0.12 | 0.28 | 3 | 1 | Phenylalanine |
| **Biosynthesis of alkaloids derived from shikimate pathway** | 0.21 | 0.46 | 7 | 3 | Shikimate, tyrosine, phenylalanine |
| **Valine, leucine and isoleucine degradation** | 1.00 | 1.00 | 5 | 1 | Leucine |
| **Inositol phosphate metabolism** | 1.00 | 1.00 | 9 | 1 | Inositol |
| **Alanine, aspartate and glutamate metabolism** | 1.00 | 1.00 | 14 | 2 | Aspartate, glutamate |
| **Pyruvate metabolism** | 1.00 | 1.00 | 5 | 1 | Pyruvate |
